# Editorial: Molecular switches of the immune system: The E-protein/Id axis in hematopoietic development and function

**DOI:** 10.3389/fimmu.2022.1062734

**Published:** 2022-11-04

**Authors:** Mikael Sigvardsson, Barbara L. Kee, Juan Carlos Zúñiga-Pflücker, Michele K. Anderson

**Affiliations:** ^1^ BKV, Linköping University, Linköping, Sweden; ^2^ Division of Molecular Hemathology, Lund University, Lund, Sweden; ^3^ Department of Pathology, The University of Chicago, Chicago, IL, United States; ^4^ Department of Immunology, University of Toronto, Toronto, ON, Canada; ^5^ Biological Sciences, Sunnybrook Research Institute (SRI), Toronto, ON, Canada

**Keywords:** E-proteins, development, lymphocyte function, innate lymphoid cells, Id-proteins

This Frontiers in Immunology Research Topic is composed by a set of review and original articles, all highlighting the roles of E-proteins in hematopoiesis.

The first evidence for a role of E-box binding (E) proteins in the immune system came from the identification of protein bound “E-boxes” in regulatory elements at the immunoglobulin locus (1, 2). The subsequent identification of these DNA binding proteins (3) revealed amino-acid homology to MyoD as well as Myc (3, 4), and created the foundation for a new research area in molecular immunology. The complexity of the regulatory networks involving these “basic-helix-loop-helix” (bHLH) transcription factors became obvious, as both broadly expressed and tissue specific family members became identified (4). These proteins are able to form homo- as well as hetero-dimeric complexes targeting E-boxes in transcription regulatory elements of both ubiquitously expressed and tissue restricted genes (4–6). Among the broadly expressed prot*ins are E12, E47 [both encoded by the E2A gene (*TCF3*) (3)], HEB (*TCF12*) (7) and E2-2 (*TCF4*) (8), that due to their overlapping activities and dimerization patterns have been denoted as E proteins [Reviewed in (9)]. In aggregate, E proteins display a high degree of redundancy, and it has been proposed that functional dose, rather than expression of any specific protein, controls developmental trajectories in hematopoiesis (10–13).

The complexity of the E protein regulatory network increased with the identification of Inhibitor of DNA binding (ID) proteins (14). The ID proteins harbor the HLH domain needed for dimerization with E proteins but lack the DNA binding basic domain (b), creating a complex that is unable to bind the target DNA elements in the genome. Thus, ID proteins are powerful functional inhibitors of bHLH protein activity with important roles in hematopoiesis. This Research Topic includes a review article describing our current understanding of the function of ID proteins blood cell development (Singh et al.). The authors describe what has been learned from gain and loss of function experiments and provide insight into the intricate interplay between the different E and ID proteins in hematopoiesis.

The importance of E proteins in lymphocyte development was revealed by the targeted inactivation of the E47/E12 encoding *E2A*. These studies revealed a critical requirement for these E proteins at the earliest stages of lymphocyte development (15–18). This Research Topic contains a review by Aubrey et al. detailing how the complex interplay between E and ID proteins drive developmental trajectories in lymphopoiesis. The article provides a molecular insight to the mechanisms by which E proteins drive lymphoid lineage differentiation and control antigen receptor recombination to generate a functional B and T lymphocytes. An original research contribution by Roels et al. focuses on a multi-omics analysis resolving the functions of E proteins in human T-cell development. This work not only provides us with insight into the evolution of the immune system but also provides information that can be explored to better understand the role of E proteins in leukemia.

The E protein/ID protein axis is also of importance for the formation of innate lymphoid cells (ILCs) (19–25). Our current understanding of the interplay between E and ID proteins in commitment to the T lymphocyte and ILC fates is the focus of the review article by Pankow and Sun presented in this Research Topic. The role of E proteins in T cell development is complex with functional importance in early development (26, 27) as well as in the generation of effector populations in the adaptive immune response (28, 29). The article by Hidaka et al highlights this complexity by reviewing the role of E and ID proteins in early T cell development as well as in the generation of regulatory T cells.

Not only TCF3 (E2A) but also TCF12 (HEB) is reported to be of essential importance for normal T-cell development (30, 31). The use of alternative *Tcf12* transcriptional start sites and alternative splicing of the transcript results in the generation of HEBCan and HEBAlt proteins. Of note, the HEBAlt protein harbors a unique N-terminal domain as compared to the HEBCan protein, and has been reported to act as a driver of early T-cell development (32, 33). In this Research Topic, an original article by Yoganathan et al. reveals that a YYY motif in the HEBAlt specific region of the protein is targeted by Janus Kinase activity. This work proposes a direct connection between E protein function and extracellular signaling events in T-cell development. Janus kinase activity is not the only signaling pathway that is functionally integrated into the E protein/ID protein axis. The review by Hwang et al. explores the signaling networks involving E- and ID proteins that control the development of T-cells as well as T-cell activation.

Among the functionally integrated signaling pathways, Notch signaling is of special interest as it is both a driver of T-cell development (34, 35) and has an important role in T-cell transformation (36, 37). Notch signaling has been reported to result in the targeting of E proteins for degradation (38, 39) suggesting that E protein dose may be directly linked to malignant transformation. The original article by Veiga et al. reports the analysis of a set of most elegant complementary T lymphocyte acute lymphoblastic leukemia (T-ALL) models revealing a unique role for TCF12 in malignant transformation. Loss and gain of function experiments provide evidence showing that a reduced TCF12 dose is redundant to NOTCH activation in the transformation process. This would indicate that targeting of TCF12 by NOTCH signaling is a major contributor to the powerful oncogenic activity exerted by NOTCH in T-ALL. In a second original article in this Research Topic, Carr et al., tested the requirements for the HMG-transcription factor LEF1, a factor previously proposed to be a component of the regulatory network involving E proteins and Notch1, in T-ALL (40). Here, they revealed that E protein deficiency promotes leukemia through adaptive mechanisms involving LEF1 addiction or independence, based on the status of LEF1 expression at the time of transformation. These papers stress the complexity by which E proteins contribute to malignant transformation, the subject of the review article by Parriott et al.. This article discusses the mechanism by which E proteins directly contribute to malignant transformation as the targets of the oncogenic bHLH proteins TAL1 and LYL1 that are overexpressed in mouse and human T-ALL.

We believe that the articles presented within the frame of this Research Topic provide timely and novel knowledge, and serve as a valuable source of information for investigators in molecular and developmental immunology.

## Author contributions

All authors listed have made a substantial, direct, and intellectual contribution to the work and approved it for publication.

## Acknowledgments

We wish to thank all authors and reviewers who has contributed to make this Research Topic a success.

## Conflict of interest

The authors declare that the research was conducted in the absence of any commercial or financial relationships that could be construed as a potential conflict of interest.

## Publisher’s note

All claims expressed in this article are solely those of the authors and do not necessarily represent those of their affiliated organizations, or those of the publisher, the editors and the reviewers. Any product that may be evaluated in this article, or claim that may be made by its manufacturer, is not guaranteed or endorsed by the publisher.
